# Recurrence Quantification of Fractal Structures

**DOI:** 10.3389/fphys.2012.00382

**Published:** 2012-10-01

**Authors:** Charles L. Webber

**Affiliations:** ^1^Department of Cell and Molecular Physiology, Stritch School of Medicine, Loyola University Chicago Health Sciences DivisionMaywood, IL, USA

**Keywords:** dynamical systems, recurrence analysis, mathematical fractals, homeodynamics, dimensionality

## Abstract

By definition, fractal structures possess recurrent patterns. At different levels repeating patterns can be visualized at higher magnifications. The purpose of this chapter is threefold. First, general characteristics of dynamical systems are addressed from a theoretical mathematical perspective. Second, qualitative and quantitative recurrence analyses are reviewed in brief, but the reader is directed to other sources for explicit details. Third, example mathematical systems that generate strange attractors are explicitly defined, giving the reader the ability to reproduce the rich dynamics of continuous chaotic flows or discrete chaotic iterations. The challenge is then posited for the reader to study for themselves the recurrent structuring of these different dynamics. With a firm appreciation of the power of recurrence analysis, the reader will be prepared to turn their sights on real-world systems (physiological, psychological, mechanical, etc.).

## Dynamical Systems in *N*-Dimensional Space

### Homeostasis versus homeodynamics

Systems, mathematical and physical, are each framed by a set of deterministic rules defined by the interaction of multiple components (variables) as coupled by adjustable constants (parameters) and scaled by fixed constants. To the extent that such systems are time-varying, they are posited to be dynamical in nature as opposed to static. Many dynamical mathematical systems are explicit, exact, noise-free, and time-reversible. But real-world systems from physics, chemistry, and biology are at best ill-defined for they exist in noisy environments and have interactions with other neighborhood systems (changing coupling strengths). The mathematical description of real-world systems is often approximate and incomplete. The presence of noise itself has the ability to shape, even tune, dynamical systems such as in the case of stochastic resonance (Wiesenfeld and Moss, 1995).

**Figure 1:**
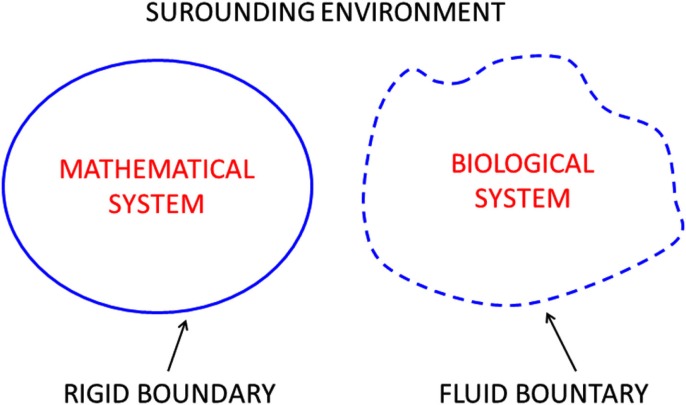
**Closed systems are distinguished from their surrounding environments by definitive boundaries, firm (left) or fuzzy (right)**.

A closed system can be conceptually portrayed as a bounded area embedded within a surrounding environment as illustrated in Figure 1. Although the simple systems are represented in two dimensions (flat), no dimensionality is implied or excluded. If the system is rigid the boundary is fixed and inflexible (solid line), but if the system is plastic the boundary (dashed line) can move and adapt to the surrounding environment. In this sense, experience teaches that the first system is more traditionally mathematical whereas the second system is more intrinsically biological. Flexibility and adaptability of the boundary determines system survival and success in harsh environments.

From the field of physiology came the very helpful concept of system homeostasis. The foundation of homeostasis stems back to Claude Bernard (1813–1878) and his concept of the *milieu intérieur* of the extracellular environment of multicellular living systems (Gross, 1998). However, it was Walter Cannon (1871–1945) who coined the term *homeostasis* (Cannon, 1929) which has since been elevated to the status of scientific law as it were. Principles of homeostasis assume that systems of the body are constrained within certain tight bounds whereby system variables are attracted to so-called constant or static values compatible with life. Good, if not obvious, examples include the control of arterial plasma pH at 7.40; normal body temperature near 37°C; and mean blood pressure around 100 mm Hg to name a few.

Homeostasis implies the presence of feedback regulation of dynamical systems affected by sensors that report back to the control center of the system. A half century ago engineering sciences started impacting physiological thinking, so much so that the concept of set points was *in vogue* for living organisms. Taken to its extreme, however, homeostasis can become a straight jacket to dynamical systems. In this context, the poster child for homeostasis might be the cadaver state where all movement is disallowed! Indeed, many living physiological systems seem to be missing an error signal (Somjen, 1992), and concepts of homeostasis and Gaussian statistics may be barriers to understanding natural variability (West, 2010).

With much deeper appreciation for the rich dynamics afforded by dynamical systems, the idea of homeodynamics is much more satisfying. Homeodynamics sets trajectories free from the overbearing constraints of homeostasis (Lloyd et al., 2001). These two concepts can be simply contrasted by considering a simple physics metaphor. Think of a system represented by a marble fallen into a hole in the center of a circular plate. With tilting motion applied to the plate (noise), the marble remains locked in its fixed position, unless the disturbance becomes too great. This is rigid homeostasis where the marble is entrapped on a strong attractor. Now think of a second system also consisting of a marble on a plate, but this plate has no center hole. As the plate is tilted motion is imparted to the marble. As long as the marble remains on the plate and moves freely over its domain, the system is stable. This is homeodynamics. Only when the tilt angle becomes too steep or the marble velocity becomes too fast does the system fail.

### Transients and non-stationarities

**Figure 2:**
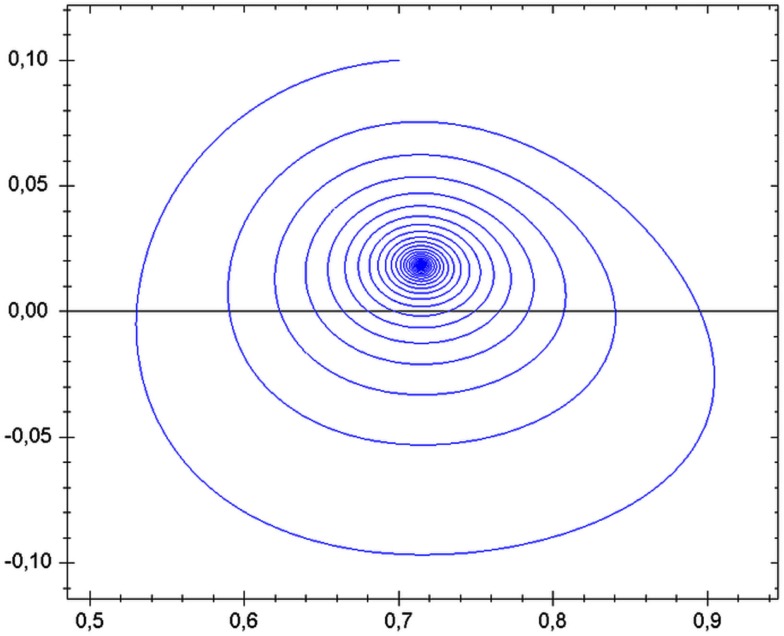
**Pathway of a transient trajectory of a dynamical system *enroute* to its stable singularity**. Public source: http://en.wikipedia.org/wiki/Phase_space

For any system to be termed dynamic, it must show motions in time or contrasts in space. The state of the system can be considered as either homeostatic or homeodynamic. Interestingly, homeostatic systems can have motions in the sense that it moves toward the well of attraction. Similar to the phase-space diagram of Figure 2, a marble swirling around a stationary funnel cone will soon come to rest in the smaller-diameter funnel spout where it will remain at its fixed point. The pathway traversed by the marble is called the trajectory and the destination is called the basin of attraction. Likewise, magnets will hold iron filings in complicated yet rigid patterns within multiple basins of attraction.

From the perspective of homeodynamics, however, trajectories can also be seen as non-stationarities operating over a field containing basins of attraction always changing. Living systems, especially, have weak and variable basins of attraction meaning that the dynamic is always on the move, never resting *per se*, as the wells of attraction rise and fall. This would be like the complex motions of a marble rolling over a large rubber sheet which was continually subjected to topological contour changes.

### Dimensionality and riemann space

**Figure 3:**
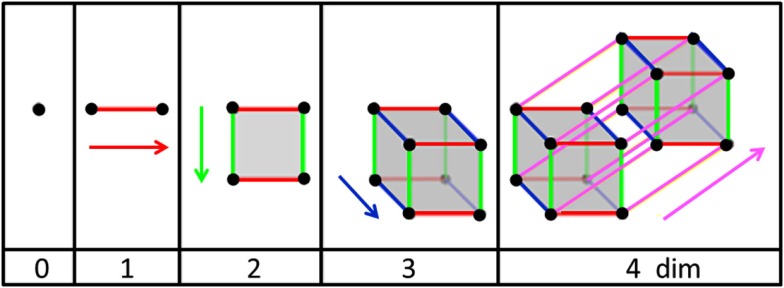
**Representation of *N* dimensions arising from points moving in orthogonal directions**. Adapted from public source: http://en.wikipedia.org/wiki/Dimension

Examine Figure 3 from left to right. If one works with points (very, very small marbles), a single point is mathematically defined as occupying a dimension of zero. As soon as a second point is introduced to the system, it must be separated by a finite distance from the first point. The line connecting the two points forms a line which resides in a dimension of 1. Sliding the horizontal line vertically defines a square (or rectangle) which lives in a two-dimensional plane (flat or curved). Shifting the square forms a cube or rectangular box which exists in three-dimensional space. Movement of the cube perpendicular to the three orthogonal axes forms a hypercube or tesseract which cannot be drawn because it exits in four-dimensional space. In this type of metric space or Riemann space, the dimensions are integers with no upper limit (0, 1, 2, 3, 4, etc.). The higher the dimension the more complex is the system that can be represented. Note that a three-dimensional system moving in time (a dimension itself) requires four variables to locate the system or object.

One definition of system complexity relates to the number of interacting variables present: the more the variables, the higher the complexity. And the higher the complexity, the greater must be the dimensionality of the system. Thus systems have dimensions that can be captured by embedding methods. For example, one can compute the distance between sub-states of the system by defining the Euclidean distance between vectors of the system. The trick is to be sure that the system is being studied in the dimension in which it resides, less it be under-represented topologically as it were. To study a three-dimensional ball in two-dimensions, a plane can cut through the object and results in a circle of varying radius depending upon where the slice is made. Conversely, to study a three-dimensional ball in four-dimensions adds no new information, but is merely a waste of computational effort. System information is maximized in the dimension in which the system lives.

There are various ways to estimate the dimension of a dynamical system, but there are two cautions to remember. First, measurement of dimensionality depends upon the system being in some kind of homeostatic steady state. This is practically realized for mathematical systems, but not necessarily biological systems. Second, the algorithms employed for the estimation of dimensionality, lose their efficiency as fast as the dimension being estimated increases. This is known supposedly as “the curse of dimensionality” in which only the lower dimensions can be measured with confidence (Parker and Chua, 1989). Third, the presence of real-world noise inflates the dimension being measured. This is not a problem for mathematical systems in which there is no noise (save digital noise), but the more the noise present the greater the real dimension of the system gets inflated. The reason for this is that no dimension exists that can completely capture the full dynamic of pure stochastic noise.

With the introduction of low-dimensional chaos (Lorenz, 1963) and fractal structures (Mandelbrot, 1983) it became fashionable to hypothesize that the complexity of biological systems could be explained by few-variable systems operating in low dimensions. But since the number of quasi-steady state experimental points required to estimate the system dimension is 10^∧^dimension, dimensions greater than six are impractical to measure and inaccurate to report. But one surprise from fractal structures was the discovery that dimensions need not be integers. Rather dimensions can be fractions (non-integers; Grassberger and Procaccia, 1983).

### Differential flows and difference maps

Time-varying (or space varying) systems in the real-world are smooth and continuous insofar that the distance from point to point is vanishingly small. Electrical analog systems best represent such continuous and smooth signals as measured as AC voltage waves from wall sockets (American: 110 V sinusoid at 60 Hz). But we live in an artificial digital world where reality is discretized into steps that are significantly larger than the vanishingly small limit in calculus. The higher the digitization frequency, the higher is the fidelity of reproduction. But magnification (amplification) of these signals always reveals the tell-tale steps of these artificial reproductions of reality.

These comments are made from a purist standpoint. However, it seems fair to declare a digitized system as continuous if (and only if) the signal is sampled at least 10 times faster than the fastest frequency within that signal. Here these quasi-smooth signals are considered to be flows, dynamical flows of the combined system variables interacting. For example, as a fly navigates a room (true continuous flow), high-frequency stroboscopic “stopping” of the motion faithfully captures the trajectory (fictive continuous flow). There is a caution here. The fair assumption above is disrupted when surprise events occur within the dynamic. No theory of maximal digitization frequency will suffice and the sampling theorem of Henry Nyquist (1889–1976) is violated (Nyquist, 1924).

**Figure 4:**
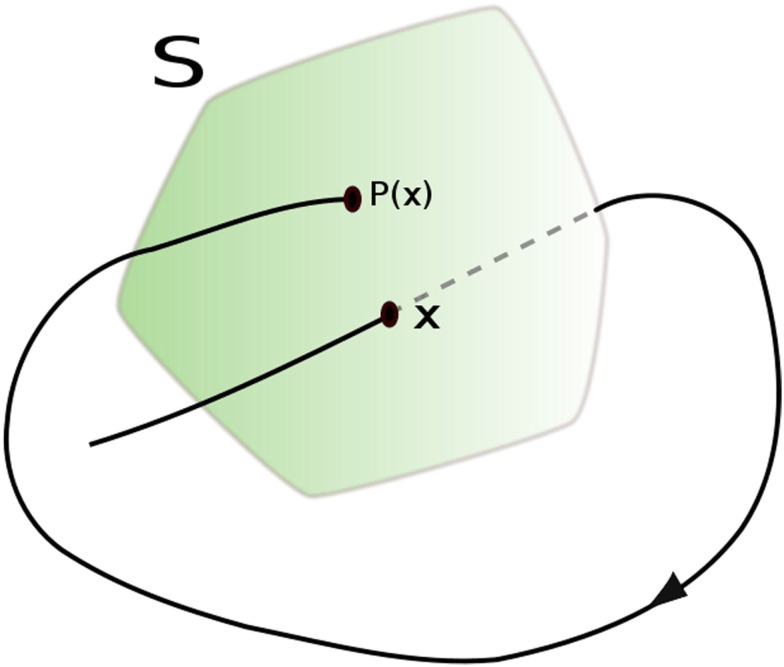
**Example of how a Poincaré section reduces the dimensionality of a dynamical system**. Public source: http://en.wikipedia.org/wiki/File:Poincare_map.svg

Another way of describing a system is to divide the continuous flow into intervals. This is particularly easy if the signal possess a stereotypic marker which can serve as triggers to end one interval and start another. Thus, interspike intervals (ISIs) are easily computed from neuronal spike trains. Likewise, R-wave to R-wave intervals (RRIs) are easily computed from the PQRST flows of the English electrocardiogram (ECG) or German *elektrokardiogramm* (EKG). In general, whether or not there are distinctive features in the time series, difference maps can still be generated by defining a barrier that is one dimension below that of the system. Mathematically these difference maps are called Poincaré sections (Rasband, 1990) named after the French mathematician Henri Poincaré (1854–1912) who contributed so much to non-linear dynamics (before the invention of the computer). Figure 4 illustrates the formation a two-dimensional Poincaré section from a smooth and continuous three-dimensional flow. Every time the dynamical flow crosses the two-dimensional surface (S), difference points are plotted on the surface. If the points in the Poincaré section form patterns, there are deterministic rules in place governing (steering) the dynamic. In this case, the next point P(*i* + 1) becomes a function of the previous point P(*i*). Such maps can diagnose simple periodicities (single point), multi-stable systems (multiple points), and chaotic trajectories (fractal points). However, if the flow is stochastic (white noise) the Poincaré section will display points in random patterns without structure, implying that no determinist rules are in place.

### Terminal dynamics

**Figure 5:**
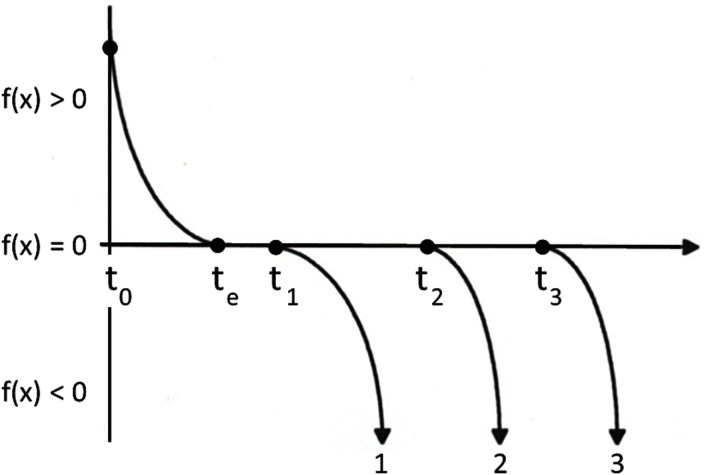
**Schematic of terminal dynamic with multiple trajectory selections**. Adapted from Figure 24.8 of Zbilut et al. (1996).

Many smooth and continuous mathematical functions are continuously differentiable and possess unique solutions of the Lipschitz type named after German mathematician, Rudolf Lipschitz (1832–1903). Other smooth and continuous mathematical functions are not continuously differentiable, have multiple solutions of the non-Lipschitz type, and are strictly non-deterministic and non-reversible in time (or space). Possible trajectories of one non-deterministic system is schematized in Figure 5. Starting at time zero (*t*_0_), f(*x*) is greater than 0 and the trajectory decays toward the horizontal axis. When the trajectory reaches this axis (*t*_e_), instead of continuing through a single point to the region of negative values (as a Lipschitz type system would do), it is extinguished (halts). Mathematically, when f(*x*) = 0 all dynamic motion ceases and the system is rendered non-Lipschitzian. The only way for the system to be kicked back into action is for infinitesimal noise to jitter the system off of this singularity, forcing f(*x*) ≠ 0. Since this dynamical action restart can happen at any time following the start of the singularity, the solutions to the equation become numerous and variable, initiating new trajectories (1, 2, or 3) where f(*x*) is non-zero at different times (*t*_1_, *t*_2_, *t*_3_, respectively). This trajectory selection is unique, flows with the direction of time, and is non-reversible through the singularity.

There are many real-world examples of non-deterministic systems which possess alternating deterministic trajectories and stochastic pauses. For example: jet aircraft land and pause at the gate before taking off again; pauses interrupt arm extensions and flexions; flatline isopotentials are recorded in the normal ECG between *T*-wave repolarizations and *P*-wave excitations; and the active running of ants is punctuated by stationary pauses. To best way to model such systems would be to describe the dynamical trajectories with differential equations interspersed with realistic (and stochastic) pauses between trajectories (system stop or pause).

## Recurrence Plots

### Auto recurrences (implemented by program RQD)

Recurrence is a theoretical mathematical concept that has practical utility in the real-world. Events can recur in time; places can be revised in space. For starters, take a time series, any digitized time series, which is by definition a linear vector of N points. Form two identical copies of this vector calling the first *V_i_* and the second *V_j_*. Compare each point in vector *V_i_* with every point in vector *V_j_* and compute the distance between them by taking the absolute differences between paired scalars according to this formula.

(1) $$\left[Dij\right]=\left|\left(Di-Dj\right)\right|\text{ for}\;i\text{ = 1}\;\text{to }N\text{and }j\text{ = 1 to }N$$

**Figure 6:**
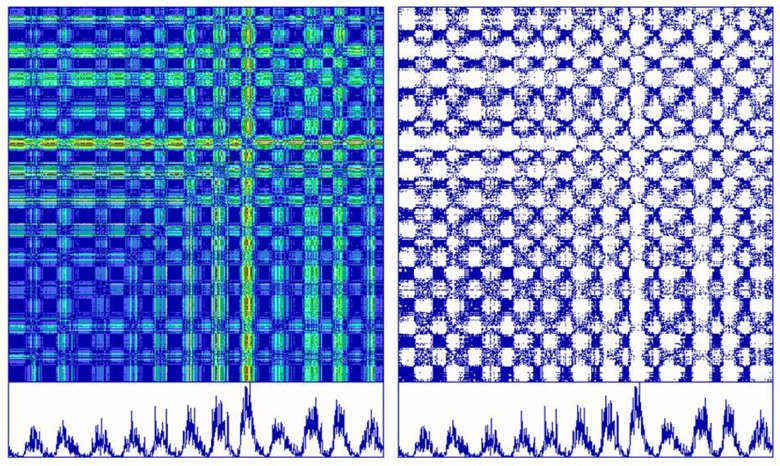
**Global (left) and local (right) recurrence plots of monthly sun spot activity from May 1874 to September 2005 (131 years, 5 months) obtained from the Royal Greenwich Observatory**. Twelve cycles of 11 years each are duplicated as time series beneath the recurrence plots. With a delay of 1 and embedding dimension of 1, the distance matrices are scaled from 0 to 100%. With the threshold set to 100% (left) the entire matrix is plotted in 10 different colors representing 10% steps (saturated, unthresholded recurrence plot). With the threshold set to 1% (right) only a fraction of the first step is plotted in a single color (sparse, thresholded recurrence plot).

This calculation will generate an [*N_i_*, *N_j_*] square matrix called the distance matrix with *N* × *N* elements. Plotting the distances at each *V_i_*, *V_j_* coordinate produces an unthresholded recurrence plot which can be color coded. A ubiquitous line of identity (LOI) forms a central diagonal where *i* and *j* scalars are always identical (distances of 0). Likewise, the distances are exactly symmetrical to around this LOI since the distance from point *i* to point *j* is the necessarily the same as the distance from point *j* to point *i*. Figure 6 (left) plots an unthresholded (global) recurrence plot that is color coded by distance (from blue = 0–10% to red = 90–100%). It can be noted that the dark blue rectangles in the recurrence plot correspond to the nadirs in the sun spot activities, but that the red recurrent points line up with the largest peak in sun spot activity.

To generate a sparse recurrence matrix, the distance matrix must be thresholded. The formula for the recurrence matrix is given below where the epsilon threshold (ε) is some fraction of the maximum distance in the distance matrix and theta (Θ) is the Heaviside function that replaces the distance matrix with either 1 for distances below threshold (close or recurrent points) or 0 for distances above threshold (distant or non-recurrent points).

(2) $$Ri,j:=\Theta \left(\varepsilon_{i}-\left|x_{i}-x_{j}\right|\right),\;i,j=1,\ldots N$$

Distance matrix thresholding is demonstrated in Figure 6 (right) in which epsilon is set to 10% of the maximum distance. In this case, only the dark blue recurrent points are plotted, leaving the remainder of the area as white space (above threshold). Thresholding converts the saturated global recurrence plot (multicolored, 100% saturated) into a sparse recurrence plot (single colored, 5.835% saturated).

**Figure 7:**
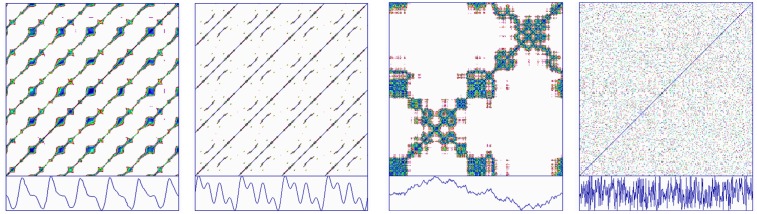
**Recurrence plots (top) of time series (bottom) including (left to right): tracheal pressure (rodent); pink noise (auto-regressive process of integrated white noise); Hénon chaotic attractor (*x* variable); white noise (caesium^137^ beta decay)**.

Typical recurrence plots from very different dynamical systems are illustrated in Figure 7. The fundamental observation is that parallel trajectories score as diagonal lines parallel to the central LOI. Periodic processes score with very long diagonals (panel 1) whereas deterministic chaotic processes score with short diagonals (panel 2). Auto-regressive processes have parallel trajectories that stack vertically, forming block patterns (panel 3). However, in the case of stochastic systems where each point in the time series is time-independent from all other points, recurrence plots lose these diagonal line structures (panel 4). Thus the key to discovering determinist rules in dynamical systems is to look for diagonal line structures, the length, number, positioning, etc. which convey insight into the organization of the dynamics.

The examples given above discuss (for simplicity) the recurrences between points. However, by implementing embedding procedures with lag delays between embedded points, it is possible to cast the dynamic into higher dimensional space. Typically, the Euclidean norm is used to compute vectors and then the Euclidean distances between all possible vectors are computed. If the distances fall below a threshold cutoff (epsilon), that vector pair is said to be recurrent. To find the proper embedding dimension, it is recommended to use the false nearest neighbor approach (Kennel et al., 1992). It is always better to overestimate the ideal embedding dimension than underestimate it so that the full dynamic can be captured in its proper dimension (as opposed to a projection to a lower-dimensional wall as it were). Then for embedding dimensions greater than 1, the proper time delay between embedded points must be found. This can be determined by looking at the first minimum in the autocorrelation function or the first minimum in the mutual information function (Fraser and Swiney, 1986). Typically, lag or delay values are greater than one for smooth flows. However, for discrete intervals (Poincaré sections of flows), lags of one works just fine.

### Cross recurrences (implemented by program KRQD)

As discussed, auto recurrence looks for parallel trajectories within a single time series. Likewise, cross recurrence looks for parallel trajectories between two time series. As explained in the equation below, the distances between all vectors pairs, *x_i_* and *y_j_* are computed and thresholded to form a recurrence matrix. In this case the LOI and symmetry across the central diagonal are both lost if *x_i_* and *y_j_* vectors are different. There are practical implementation rules for computing cross recurrence plots (Webber, 2012). First, both signals must be digitized simultaneously at the same digitization rate. Second, both signals must be amplitude adjusted over the same range (e.g., the unit interval from 0 to 1) to minimize the distance between parallel but separated trajectories. Third, the signals must be smooth flows, not discrete intervals. Fourth, the lag intervals should be set to 1. Cross recurrence plots are useful in separating out events in one signal that lead, lag, or occur simultaneously with the second signal.

(3) $$Ri,j:=\Theta \left(\varepsilon_{i}-\left|x_{i}-y_{j}\right|\right),\;i,j=1,\ldots N$$

**Figure 8:**
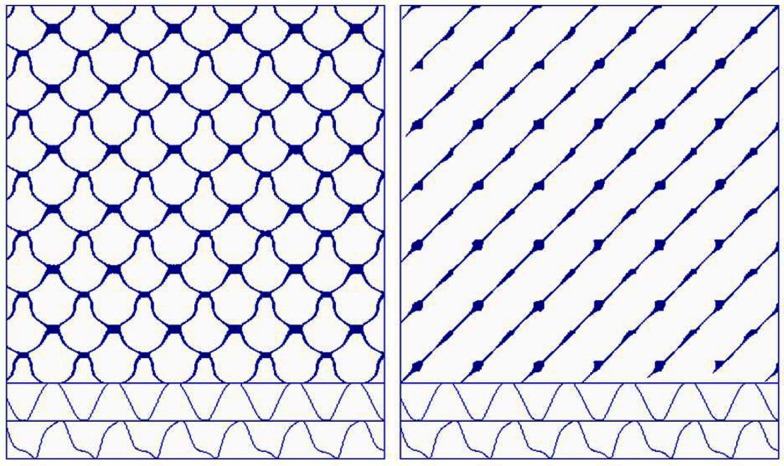
**Cross recurrence plot (left) and joint recurrence plot (right) of a fluid-coupled system consisting of an independent sinusoidal driver (upper trace) and dependent coupled rotor (lower trace)**.

Shockley et al. (2002) performed coupled-oscillator experiments on a fluid dynamical system. A gravity-driven rotor was freely spun within a tray filled with a fluid of selectable viscosity (low, medium, or high). Then the tray was pushed and pulled horizontally by a sinusoidal driver motor system. As shown in Figure 8 (left) for a high viscosity medium, the sinusoidal motion of the driver tray distorted the motion of the rotor (lower two traces). The non-linear coupling of the rotor to the driver was then studied by cross recurrence plots which in this case shows the high degree of non-linearity along deterministic squiggles which form crossing patterns (due to the embedding dimension being selected as 1).

### Joint recurrences (implemented by program JRQD)

The concept of joint recurrences is different from that of cross recurrences (Marwan et al., 2007). That is, instead of looking for parallel trajectories between two time series, joint recurrences look for recurrent points common to the auto recurrence plots of each signal separately. By this means joint recurrences can detect direction of phase synchronizations. Joint recurrence plots are expressed mathematically as the intersection of two individual auto recurrence plots of separate time series, *x* and *y*. It is advised that recurrence parameters be selected the same for each time series, but this is not absolutely necessary theoretically. The utility of joint recurrence plots awaits further exploration.

$$\begin{array}{llll} & Ri,j:=\left[\Theta \left(\varepsilon_{i}-\left|x_{i}-x_{j}\right|\right)\right]\;\cap \;\left[\Theta \left(\varepsilon_{i}-\left|y_{i}-y_{j}\right|\right)\right] & & \\ & \;i,j=1,\ldots N & & \text{(4)} \end{array}$$

Returning to the coupled-oscillator experiments of Shockley et al. (2002), the coupling of rotor to driver were reexamined by joint recurrence plots. In this case, Figure 8 (right) shows the high degree of linearity along deterministic lines with bulges at periodic intervals. Taken together, this simple example highlights how joint-recurrences and cross recurrences are two different ways in which coupled system variables can be studied. (More work is required in this area.)

## Recurrence Quantifications

The recurrence plot when first reported was heralded as a mathematical tool for revealing hidden rhythms within complex time series (Eckmann et al., 1987). And so it is. But it soon became apparent that recurrence plots had two inherent difficulties. First, there were numerous recurrence parameters that needed to be set logically to match the data set under investigation. These parameters included the threshold radius, embedding dimension, time delay between embedded points, selection of the distance norm (max, min, or Euclidean norm), rescaling of the distance matrix, a parameter that defined the shortest number of recurrent points forming a line segment (typically two), and the size of the recurrence window) short, medium, long. The author has written extensively on how to select recurrence parameters elsewhere (Webber and Zbilut, 2005).

The second difficulty with recurrence plots was the plots themselves. That is, how are the intricate and beautiful patterns to be interpreted? Instead of reading into recurrence plots patterns unique to the observer (I see a canoe in the clouds, you see a banana), recurrence quantification were born to extract from recurrence plots different aspects of the plots. To date there are eight unique features that are extracted from recurrence plots according to strict mathematical definitions. These recurrence variables, as they are called, include *percent recurrence* (recurrence density or recurrence rate), *percent determinism* (portion of recurrent points aligning into diagonal lines), *d*_max_ (length of longest diagonal line), *Shannon entropy* (complexity of line structure distributions), *trend* (homogeneity or inhomogeneity of recurrent points over plot), *percent laminarity* (portion of recurrence points aligning into vertical lines), *v*_max_ (length of longest vertical line), and *trapping time* (average vertical line length). The strict mathematical definition of these variables can be found elsewhere (Webber and Zbilut, 2007; Webber et al., 2011). The idea is that from a single time series (with auto recurrence plots) or double time series (with cross or joint recurrence plots) multiple reporters of the embedded dynamic are produced. It is the differential sensitivities of these recurrence variables depending upon the system under study that render RQA as a sensitive non-linear, multidimensional tool for exploring the so-called hidden rhythms in complicated signals. The beauty of this analysis is that no modeling assumptions on the time series are required, no statistical distributions are excluded, inherent noise in the signal does not stymy the analysis since the threshold is adjustable, short data sets (*n* = 30) can yield useful data, and outliers need not be dropped, clipped, substituted, or replaced.

## Mathematical Fractals

### Fractals and recurrence structures

There is a natural linkage between fractals and recurrence. By definition, fractals are self-similar structures observed repeatedly or recurrently at different scales of magnitude. The natural world is filled with fractal examples such as mountains, clouds, trees. Thus small trigs from real trees conveniently pass as surrogates for full trees on HO train layouts. In physiology, the lungs form a fractal branching pattern from trachea to terminal alveoli with 23–27 branched generations. This fractal form minimizes the dead space volume of the conducting pathways (airways without alveoli), delivers oxygenated air quickly to the live space (airways with alveoli), and packs the entire lung within the thoracic space yet provide a huge surface area for gas exchange (70 m^2^). Likewise, the human circulatory system starting with the large single aorta (2.5 cm diameter) branches numerous times to finally form the millions of tiny systemic capillaries (7 μm diameter), only to collect them again from venules to vena cava and to repeat (recur) the process once again in the pulmonary circuit flowing through the lungs. The bronchiolar tree for air flow is also fractal and amenable to fractal modeling (Canals et al., 2004).

The theoretical study of fractals comes from the field of mathematics. Unlike natural fractals which have fundamental limits or minima (e.g., patent alveoli cannot be smaller than 100 μm and capillaries with blood flow cannot be smaller than 5 μm), mathematical fractals are infinitely deep and unbounded. From the study of mathematical fractals comes the concept of self-similarity in which graphical depictions of systems at deep levels reveals (reflects, recurs) images of larger parent structures magnitudes of scales distant. And these are not simple structures of geometric forms, but complex structures of lace-like beauty waiting to be discovered using the computer as a digital microscope.

Mandelbrot (1924–2010) is the father of fractal geometry. He is famous for asking the question, “How long is the coast of Britain?” (Mandelbrot, 1967). The answer to this question is, surprisingly, the cumulative length depends upon the length of the measuring ruler! That is, the shorter the ruler, the longer is the measured length. The longest total length would be the integral of the boundary taken to the infinitesimal limit. The conclusion is that the quantitative description of structures is scale dependent. Because of this fact, Mandelbrot was able to construct artificial worlds from algorithmic computations on the computer that could pass as actual geography in the real-world. It is not overstating the situation to affirm that our natural world is not as geometric as it is fractal in design.

What follows are detailed descriptions of five mathematical fractals, the dynamics of which can be studied by recurrence strategies. The first two fractal systems are continuous flows, and the remaining three fractal systems are discontinuous maps. Sufficient information will be provided to allow the reader to study the dynamics of these fractals in detail. More questions will be raised than answered, but the intent of the author is to simulate further research into this fascinating field linking mathematical fractals with recurrence plots and quantifications. The methodology is fully applicable to fractal biological systems (Bassingthwaighte et al., 1994) which are not addressed herein because of space constraints.

### Lorenz attractor

Edward Lorenz (1917–2008) was an American mathematician and meteorologist who studied a simplified model of atmospheric convection using three ordinary differential equations. The way air swirled around the overhead atmosphere depended on three parameters (*a*, *b*, *c*) which dictated the interaction of three strongly coupled variables (*x*, *y*, *z*) as defined below.

$$\begin{array}{ll} \text{d}x/\text{d}t=a\times (y-x) & \text{(5)}\\ \text{d}y/\text{d}t=-b\times x-y-x\times z & \text{(6)}\\ \text{d}z/\text{d}t=-c\times z+x\times y & \text{(7)} \end{array}$$

where:

*a* = 10 (ratio of the fluid viscosity of a substance to its thermal conductivity).

*b* = 28 (difference in temperature between the top and bottom of the gaseous system).

*c* = 8/3 (width to height ratio of the box being used to hold the gaseous system).

and

*x* = rate of rotation of convection cylinder.

*y* = temperature differential at opposite sides of the cylinder.

*z* = deviation of the system from a linear, vertical graphed line representing temperature.

**Figure 9:**
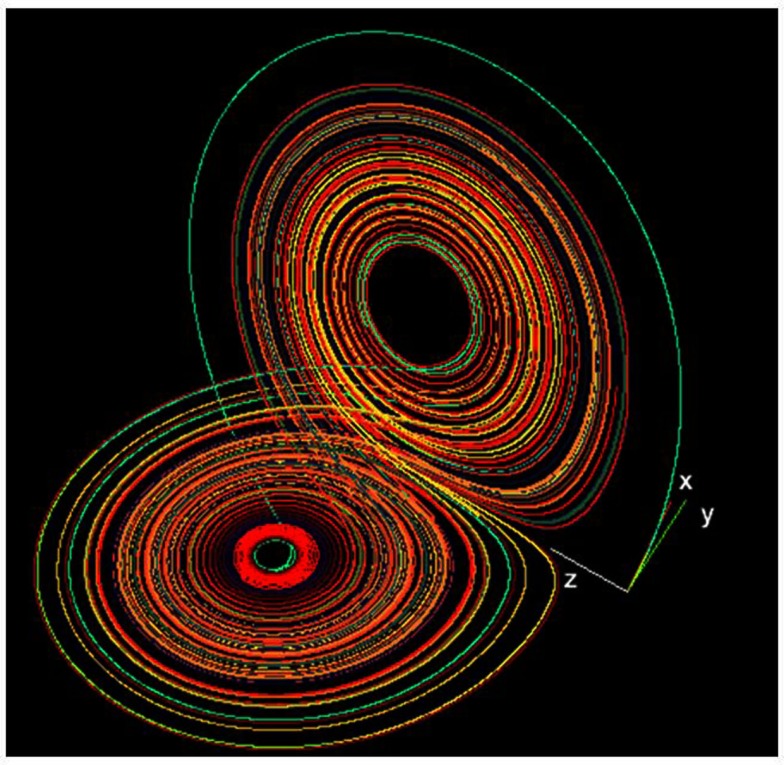
**The Lorenz strange attractor in its chaotic mode**. Public source: http://en.wikipedia.org/wiki/Lorenz_system

Mathematical solution of the system of Lorenz equations results in a three-dimensional structure known as a dynamical attractor. Dynamical motion is captured on the single trajectory forming the attractor, but other negative spaces are devoid of legal trajectory pathways. The contrast between the presence and absence of trajectory pathways gives shape to the attractor which appears like the wings of a butterfly (with asymmetric donut holes) as shown in Figure 9.

What is remarkable about the Lorenz attractor (and consequently a fundamental principle of chaotic dynamics) is that not only can rich dynamics be continued within and expressed by simple non-linear systems (e.g., consisting of a mere three variables), but that the single dynamical trajectory shows sensitive dependence on initial conditions. What this means is that by just altering the initial conditions of just one variable by a smidgeon (non-mathematical term) will lead to two different trajectories over time. For example, if variables *x*_1_ and *x*_2_ differ numerically by just 10^−5^ (*x*_1_ = 0.10000 and *x*_2_ = 0.10001), everything else remaining exactly the same, then the pathways will eventually diverge. Such extreme sensitivity of chaotic systems to initial conditions has been called the “butterfly effect” in honor of insect shape of the Lorenz attractor.

To study the recurrence structure of the Lorenz system, the three system variables can be followed over time by solving the three ordinary differential equations using standard fourth-order Runge–Kutta estimations. Initial conditions can be set as variables *x* = *y* = *z* = 0.1 using fixed parameters *a* = 10, *b* = 28, and *c* = 8/3. The first points of the three-dimensional trajectory can be retained to follow the transients (off-attractor dynamics) before the dynamic settles on the attractor proper. The higher the time increment (e.g., d*t* = 0.01), the longer will be the transient. The Lorenz attractor can be viewed in the *x*,*y* plane (two paper plates), the *x*,*z* plane (butterfly), and the *y*,*z* plane (owl mask) which are projections of the three-dimensional object.

Since the Lorenz attractor is a three-dimensional structure, auto recurrence plots can be generated on any one of the variables. Selecting an embedding dimension of 3 will suffice because the dimension of this attractor is fractal between 2 and 3. And any two variables can be paired to generate cross recurrence plots (KRQD *x* *y*; KRQD *x* *z*; KRQD *y* *z*) or joint recurrent plots (JRQD *x* *y*; JRQD *x* *z*; JRQD *y* *z*). See Webber (2012) for free RQA software and detailed explanations of proper implementation procedures.

### Rössler attractor

Otto Rössler (1940–present) is a German biochemist responsible for the mathematical attractor that bears his name. The Rössler attractor is similar to the Lorenz attractor and consists of three coupled differential equations. Insofar that the first two equations are linear, the Rössler attractor turns out to be simpler than the Lorenz attractor and easier to analyze.

$$\begin{array}{ll} \text{d}x/\text{d}t=-y-z & \text{(8)}\\ \text{d}y/\text{d}t=x+a\times y & \text{(9)}\\ \text{d}z/\text{d}t=b+z\times (x-c) & \text{(10)} \end{array}$$

where:

*a* = 0.2, *b* = 0.2, and *c* = 5.7.

**Figure 10:**
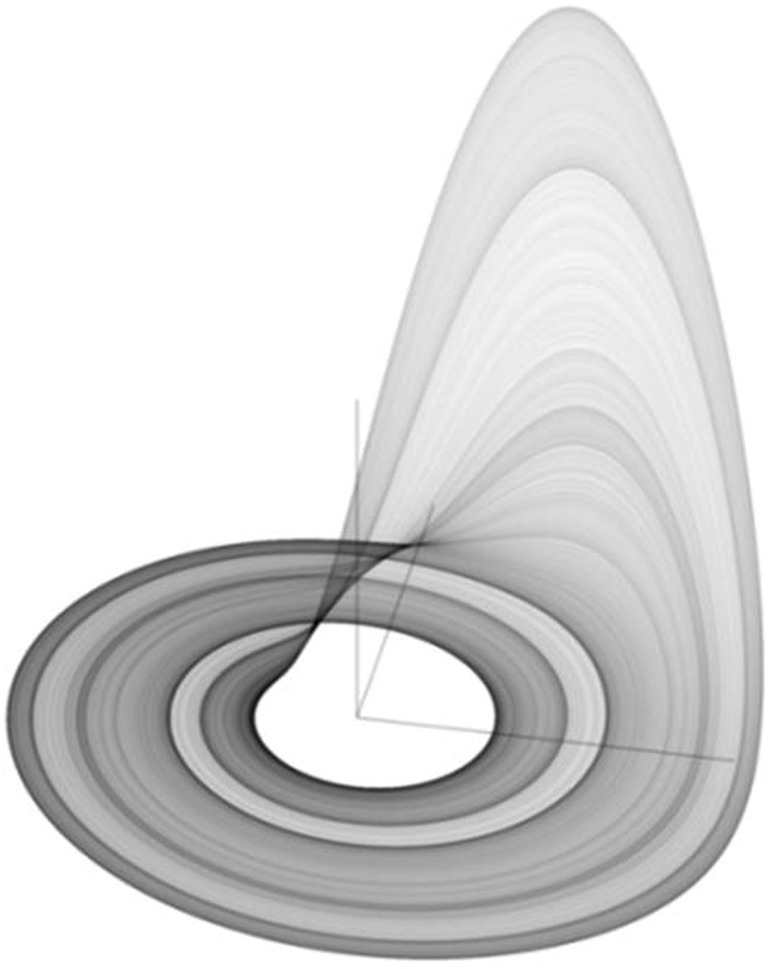
**The Rössler strange attractor in its chaotic mode**. Public source: http://en.wikipedia.org/wiki/R%C3%B6ssler_attractor

As shown in Figure 10, the Rössler attractor is a three-dimensional chaotic attractor, with unstable spiral orbits in the *x*,*y* plane that grow up into the *z* plane. The system has only one manifold and is fractal in nature. Like the Lorenz attractor, the Rössler attractor demonstrates sensitive dependence on initial conditions, the hallmark of chaos, and fracticality.

To study the recurrence structure of the Rössler system, the three system variables can followed over time by solving the three ordinary differential equations using a standard fourth-order Runge–Kutta estimation. For example, starting conditions can begin with variables *x* = *y* = *z* = 0.1 using fixed parameters *a* = 0.2, *b* = 0.2, and *c* = 5.7. Again the initial points the three-dimensional trajectory (transient or off-attractor dynamics) can be studied as can the following points (stable or on-attractor dynamics) using a high resolution time increment of d*t* = 0.01. The *x*, *y*, and *z* variables can be examined using recurrence plots and quantifications.

As can be demonstrated for the Lorenz attractor, the Rössler attractor can be shown to possess steady state, periodic, and chaotic dynamics depending up the value of the *b* parameter. As parameter *b* decreases from 2 to about 1.44, the *x* variable settles on single point attractors. With further decreases in parameter *b*, the *x* variable falls into a period-2 then period-4, then period-8 stable periodic states until full chaos erupts with *b* values less than 0.7. There are brief periodic windows embedded within chaotic regimes for lower values of b approaching 0. But at *b* = 0.2 as is the typical choice, the Rössler attractor is in a strong chaotic mode.

### Logistic attractor

Robert May (1938–present) called attention to the logistic map (May, 1976). This deceptively simple difference equation illustrates how complex dynamics can arise from non-linear recurrent interactions of a single variable. In this case, the next *x* is a function of the current *x*^2^ term.

(11) $$X_{n+1}=a\times X_{n}\times \left(1-X_{n}\right)$$

where:

*a* = 0–4 and 0 ≤ *x* ≤ 1.

**Figure 11:**
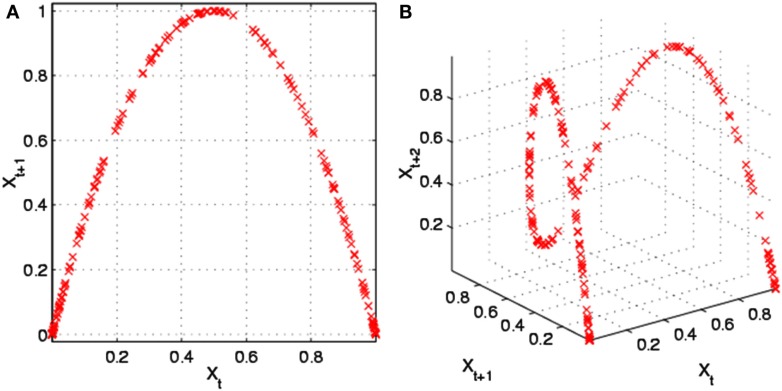
**The Logistic map in two-dimensions (A) and three-dimensions (B)**. Public source: http://en.wikipedia.org/wiki/File:Logistic_map_scatterplots_large.png

As illustrated in Figure 11, the logistic map forms a “Saint Louis Arch” in the second dimension (Figure 11A) and a “roller coaster” in the third dimension (Figure 11B). Interestingly, as tuning parameter *a* is increased from 0 to 4, the dynamics of *x* follows a period-doubling pathway to chaos. Trulla et al. (1996) investigated these dynamics with RQA windows by adiabatically incrementing parameter *a*. Transitions between periodicity and chaoticity were easily distinguished by RQA variables, particularly DET and LMAX. In fact, 1/LMAX values positively correlated with Lyapunov exponents in the chaotic frames, confirming the postulate of Eckmann et al. (1987).

The principle difference between the Logistic attractor and the Lorenz and Rössler attractors is that it is an iterated map, not a continuous flow. Because sequential points are iterated, they resemble Poincaré sections not unlike how R–R intervals represent planes through the ECG flow dynamic. In any case, it is proper to select a delay of one point when dealing with iterated dynamics (difference equations as opposed to differential equations).

### Hénon attractor

Another excellent example of an iterated map is the Hénon attractor named after French mathematician, Michel Hénon (1931–present). As explicitly defined in the equations below, this system consists of the interplay between *x* and *y* variables interlinked through two parameters, *a* and *b*, and a single constant, 1. The non-linearity of this two-dimensional system derives again from the *x*^2^ term.

$$\begin{array}{llll} & X_{n+1}=y_{n}+1-a\times X_{n}^{2} & & \text{(12)}\\ & Y_{n+1}+b\times X_{n} & & \text{(13)} \end{array}$$

where:

*a* = 1.4 and *b* = 0.3.

**Figure 12:**
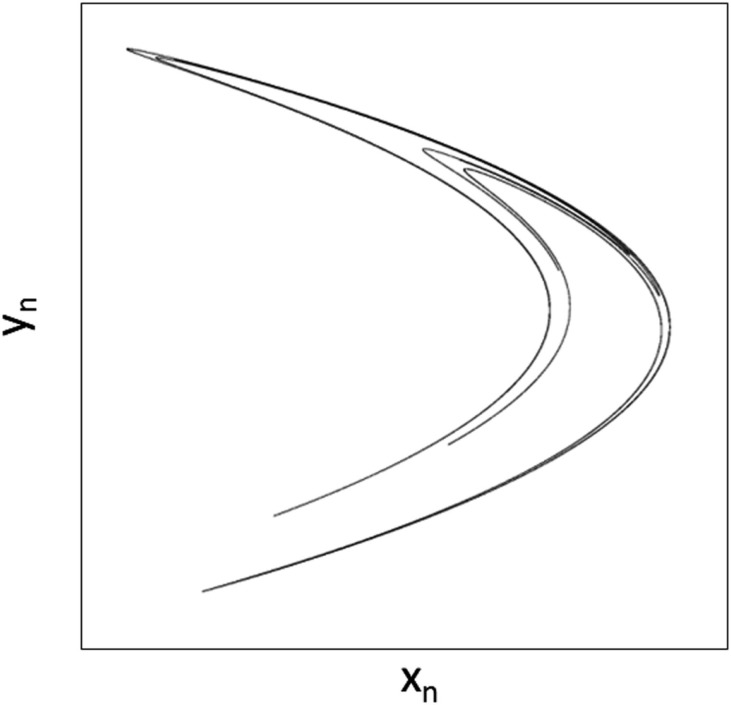
**The Hénon strange attractor in its chaotic mode**. Adapted from public source: http://en.wikipedia.org/wiki/H%C3%A9non_map

The plane plot of the coupled *x*,*y* variables reveals the double crescent shape of the Hénon attractor as shown in Figure 12. Dark points show allowed positions of the dynamic, and white space reveals disallowed positions never part of the stable dynamic. Transients can be studied by examining the *x*,*y* variables starting at randomly selected initial conditions for *x*_0_, *y*_0_. The fracticality of the Hénon attractor can be demonstrated by focusing in on one of the single arms of the double crescent (magnify the scale) and discovering yet another double-banded structure. This unveiling of bands after band continues to infinity!

It is very instructive to study the dynamics of the Hénon attractor by following the time courses of *x*, *y* individually and coupled in both the chaotic and periodic modes of the system. To get started, hints can be gleaned from Webber and Zbilut (1998).

### Mandelbrot attractor

Mandelbrot has already been introduced above as the father of fractal geometry. However, most interesting and beautiful is the mathematical set named after him. The Mandelbrot set is a flat structure with infinitely deep fractal patterns that lives in the complex plane. The intriguing and early book, *The Beauty of Fractals*, by Peitgen and Richter (1986) captures much of the essence of the Mandelbrot Set which stems from the simple iteration of the complex equation consisting of one complex variable, *z*, and one complex constant, *c*. The non-linear chaotic dynamics of this equation grows out of the complex and real parts of both the variable *z* (*z* real, *z* imaginary) and the constant *c* (*c* real, *c* imaginary), the former of which is squared according to the following equation.

(14) $$z_{n+1}=z_{n}^{2}+c$$

**Figure 13:**
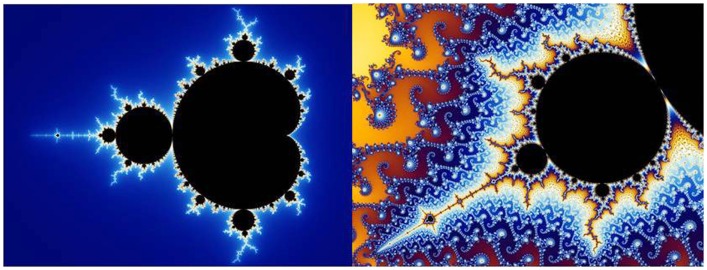
**Mandelbrot set low resolution (left) and high resolution (right)**. Public source: http://en.wikipedia.org/wiki/File:Mandel_zoom_00_mandelbrot_set.jpg

The Mandelbrot set (M set) is a black and white set meaning that complex point c either belongs to the M set (black) or does not belong to the M set (white). To keep things simple, the equation can be implemented by setting both *z* real and *z* imaginary to zero and setting *c* real from −2 to +1 and *c* imaginary from −1 to +1. Iteration of the equation will alter the *z* variable to either some type of converging dynamic (period 1, 2, 4, 8, etc. or chaotic) or diverging dynamic (tending toward infinity). If the system converges then constant *c* is a member of the M set and can be plotted as a black point on the complex plane of *c* imaginary versus *c* real. If the system fails to converge than constant *c* is a not a member of the M set as is plotted as a contrasting white point. The M set is illustrated in Figure 13 in which characteristic cardioids are seen at both low and high magnifications, demonstrating the fractal structuring of the set. Mandelbrot conjectured that his set was discontinuous, meaning the some white space interspersed between points of the set. But Douady and Hubbard (1984/1985) proved that the M set was truly continuous. To visualize the continuity of the set requires high resolution computer graphics.

To illustrate recurrence properties of the M set, formula 14 was iterated 1000 times using as initial conditions: *z*_real_ = 0.0, *z*_imag_ = 0.0, *c*_real_ = −0.75, and *c*_imag_ = 0.005. In the c plane this is positioned deep within the seahorse valley, the gap between the large cardioid to the right and smaller circle to the left (Figure 13, left). The question is, is this specific point a member of the M set or not? In this case, variable *z* was iterated 632 times before it started going toward infinity. Thus complex point *c* is not a member of the M set, but still it took many iterations to determine this.

**Figure 14:**
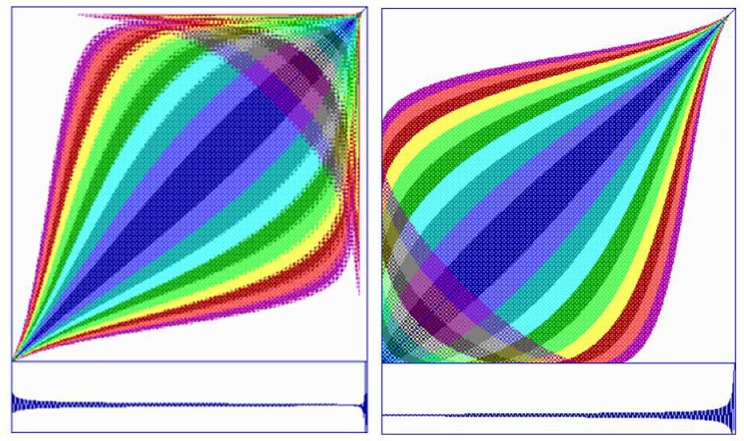
**Recurrence plot of *z*_real_ (left) and *z*_imag_ (right) scaled from point 1 to point 629 or the 633-point time series (lower traces)**. The system diverges toward infinity at the end of the each series, but the dynamics of the real and imaginary components are rather different.

The dynamics of *z*_real_ and *z*_imag_ were studied individually by generating their respective recurrence plots as shown in Figure 14. Using a delay of one, embedding of five, no rescaling of the distance matrix, and absolute radius of 0.01 and color steps of 0.001 (dark blue to purple). It can be noted that *z*_real_ shows a gentle decrescendo in terms of amplitude whereas *z*_imag_ displays a gradual crescendo. Nevertheless, after 632 iterations the system explodes toward infinity. Thus imaginary point *c* is very close to the M set border, but never touches it.

The reader is challenged to study other transient dynamics of the complex *z* variable as it moves from 0.0 to either a steady state dynamic or a non-steady state dynamic depending upon the value of complex parameter *c*. The most complicated and most interesting dynamics are seen at the borders at very high magnifications deep within the M set. In these places the *c* parameter is taken out to the sixth decimal point or finer causing the *z* variable to go through hundreds of iterations before it diverges or converges. Here the user can examine *z* real and *z* imaginary variables either individually or coupled using recurrence programs. No space remains in this paper to carry this out, so the reader should take it as an assignment to discover the rich dynamics of the iterated equation in which are hidden all the exquisite beauty of the M set.

## Conclusion

In this communication we have moved from (1) conceptual definitions of systems to (2) simple overview themes of recurrence quantifications for analysis of non-linear (and linear) systems to (3) practical implementation of recurrence analyses on systems of common fractals. By design (space limitations notwithstanding) much work has been left to the reader for study fractals on his/her own by combining these conceptual and practical ideas. For the experienced RQA user, it will be easy to move into the mathematical fractal world using recurrence strategies. For the new RQA user, it will be absolutely necessary to first read the long chapter (monograph) written by the author to learn the proper procedures for setting RQA parameters and interpreting RQA variables (Webber and Zbilut, 2005). Learning by doing is always the best teacher.

Deemphasized in this chapter is the specific application of recurrence plots and quantifications to real-world systems found in physics, chemistry, biology, and medicine, for example. The author has already addressed these things elsewhere (Webber and Zbilut, 2005). The value of this present chapter is to identify fractals as mathematical systems which possess deep-rooted complexity and repeating structures at different magnification scales. In this sense they become analogies for real-world systems which possess many of the same properties. Whether a system be mathematical or material, it is governed by dynamical rules which define boundaries, fuzzy or sharp, depending upon the state of the system (quasi-steady state or transient), and the presence of noise (numerical round-off or environmental).

The big idea of this chapter is that dynamical rules in complex, non-linear systems can be ferreted out as it were, by applying recurrence analyses to dynamical time series. Embedding procedures allow measured variables to serve as surrogates for unmeasured variables (Webber and Zbilut, 2005). The reader is challenged to apply RQA to systems of their choice. We live in a fractal world, nay fractal universe. And recurrence analysis is one way to delve into the mysteries which lie before us.

## Conflict of Interest Statement

The author declares that the research was conducted in the absence of any commercial or financial relationships that could be construed as a potential conflict of interest.
